# Erythritol, a Non-Nutritive Sugar Alcohol Sweetener and the Main Component of Truvia®, Is a Palatable Ingested Insecticide

**DOI:** 10.1371/journal.pone.0098949

**Published:** 2014-06-04

**Authors:** Kaitlin M. Baudier, Simon D. Kaschock-Marenda, Nirali Patel, Katherine L. Diangelus, Sean O'Donnell, Daniel R. Marenda

**Affiliations:** 1 Department of Biology Drexel University, Philadelphia, Pennsylvania, United States of America; 2 Department of Biodiversity, Earth and Environmental Science, Drexel University, Philadelphia, Pennsylvania, United States of America; 3 Department of Neurobiology and Anatomy, Drexel University College of Medicine, Philadelphia, Pennsylvania, United States of America; AgroParisTech, France

## Abstract

Insecticides have a variety of commercial applications including urban pest control, agricultural use to increase crop yields, and prevention of proliferation of insect-borne diseases. Many pesticides in current use are synthetic molecules such as organochlorine and organophosphate compounds. Some synthetic insecticides suffer drawbacks including high production costs, concern over environmental sustainability, harmful effects on human health, targeting non-intended insect species, and the evolution of resistance among insect populations. Thus, there is a large worldwide need and demand for environmentally safe and effective insecticides. Here we show that Erythritol, a non-nutritive sugar alcohol, was toxic to the fruit fly *Drosophila melanogaster*. Ingested erythritol decreased fruit fly longevity in a dose-dependent manner, and erythritol was ingested by flies that had free access to control (sucrose) foods in choice and CAFE studies. Erythritol was US FDA approved in 2001 and is used as a food additive in the United States. Our results demonstrate, for the first time, that erythritol may be used as a novel, environmentally sustainable and human safe approach for insect pest control.

## Introduction

Insects have significant worldwide deleterious impact on human health, agriculture, and economic growth [1]. Cost of application of insecticides for the prevention of insect damage has been estimated at $10 Billion annually in the US alone [2]. Further, widespread use of toxic insecticides continues to pose a significant threat to human health, as highlighted by recent deaths in Bihar India [3]. Thus, there is a strong need for cost-effective and human-safe insecticides to control insect pest populations.

During an examination of the effects of commonly used non-nutritive sweeteners on the longevity of *Drosophila melanogaster*, we discovered that erythritol, the main component of the sweetener Truvia, was toxic when ingested by fruit flies as compared to similar concentrations of nutritive sugar controls (sucrose, corn syrup) and other non-nutritive sweeteners. We describe here the effects of erythritol and Truvia on the longevity and motor function of the fruit fly, *Drosophila melanogaster*. We show that when flies consumed erythritol their longevity decreased in a positive concentration-dependent manner. We also use choice tests and capillary feeding (CAFE) assays to show that flies consumed erythritol when given free access to control (sucrose) food sources and suffered decreased longevity. Consumption of erythritol is safe to humans, even when consumed at high levels [4], [5]. Thus, we suggest erythritol has potential for use as a novel, human-safe insecticide.

## Materials and Methods

### *Drosophila* culturing and sample sizes for solid food studies

All animals were cultured at 25°C, kept at 50–60% humidity, and were raised under a standard 12∶12 light dark cycle. For each experimental treatment n = 30 flies were tested in groups of 10 flies per tube, and three tubes per treatment. In each treatment one tube contained males, one females, and one tube contained five flies of each sex. Tubes were kept on their side to minimize subjects becoming mired in the food. Foods were replaced twice a week. The total number of fruit flies used for these experiments was 690, with 300 used for two initial trials testing mortality among store-brand sweeteners, 120 used for repeating this with sweeteners with blue dye (0.05%) and pure erythritol, 120 for choice trials and 150 for concentration trials.

Standard *Drosophila* food for larval culturing consisted of water, cornmeal, yeast, molasses, and agar, as previously described [6]. A similar food (without molasses) also served as the base to which treatments were added. The addition of cornmeal and yeast assured the flies still received sufficient carbohydrates and protein in addition to any effects of the treatment additives. We combined *Drosophila* food with an equal weight/volume (0.0952 g/ml) of one non-nutritive sweetener (Truvia, Equal, Splenda, Sweet'N Low, or PureVia) or a control nutritive sweetener (controls: sucrose or corn syrup). We initially raised wild type (Canton S) larvae on the standard food and transferred 0–24 hour old adult flies to foods containing one non-nutritive sweetener or a control treatment and observed them for 65 days. Longevity assays and climbing behavioral assays were performed as previously described [6]. The number of dead flies were scored daily. Climbing behavior was assayed every second day. For climbing assays, a modified version of Le Bourg and Lints was used [7]. Groups of 10 or fewer flies were transferred to a clean, empty vial and given 18 seconds to climb 5 centimeters. The number of flies that successfully reach the 5 centimeter line were recorded. We compared the longevity of flies raised on food containing an equal weight/volume (0.0952 g/ml) of each of these sweeteners to control foods. Experimenters were blinded to treatments when assessing mortality and climbing ability. The exception was corn syrup, as it is not a white solid and can therefore be texturally discerned. This procedure was repeated with foods containing brilliant blue FCF (Fisher 50-727-25) in 0.05% weight/volume concentration [8], as well as erythritol, sucrose, Truvia, or PureVia as treatments. Flies were then examined daily for externally visible blue guts for 14 days. The number of dead flies and blue flies were scored daily.

### Concentration Trials

We pepared standard fly foods as previously described, then added treatments of 2 M, 1 M, 0.5 M and 0.1 M concentrations of erythritol and 0.5 M sucrose control. We placed 0 to 24 hour-old *Drosophila* on these foods and recorded mortality daily for 35 days as above.

### Choice Experiments

We prepared foods containing 2 M erythritol, 1 M erythritol and 1 M sucrose for paired presentations in open choice tests. In each treatment one food type contained 0.05% brilliant blue FCF (Fisher 50-727-25). The blue dye allowed visual confirmation of feeding on the dyed food in the pair. We presented the flies with access to two food choices by using a modified cotton stopper with approximately a 1.5 centimeter diameter hole to connect each pair of food tubes. We set up three choice trial groups: the first was between blue 1 M erythritol and non-blue 1 M erythritol foods (blue guts would confirm the blue dye did not completely inhibit feeding and confirm erythritol was being consumed), the second was between blue 1 M erythritol and non-blue 1 M sucrose foods (blue guts would confirm confirm erythritol was being consumed in the presence of sucrose), and the third was a choice between blue 1 M sucrose and non-blue 1 M sucrose, as a negative control (blue guts would confirm the blue dye did not inhibit feeding). The final choice treatment was between blue 2 M erythritol food and non-blue 1 M sucrose food (this treatment provides a comparison with the 1 M erytritol/1 M erythritol treatment as a test of dilution of toxicity by alternative food sources; blue guts would confirm confirm erythritol was being consumed in the presence of sucrose). We recorded number of flies with visible blue gut contents and mortality daily for 30 days.

### CAFE experiments

CAFE experiments were performed as described in [9]. Briefly, flies were held in vials with a cheesecloth bottom over water to promote high humidity; the vials were plugged with cotton and liquid food was presented using microcapillaries. Ten Canton S flies aged 0–12 hours old were used per vial. We used one vial of males and another of females for earch treatment. The experiments were conducted in a room that was kept at 25°C and 50–60% humidity. We made solutions of 5% w/v sucrose and erythritol in water, with 0.05% brilliant blue FCF dye added (Fisher 50-727-25). Each treatment solution was loaded in a 5 µl calibrated glass microcapillary tube (VWR International 53432-706) and inserted through the cotton plug on the vial such that the bottom of each capillary was accessible to the flies. We simultaneously held one control capillary of each solution in a similar CAFE apparatus without flies to quantify liquid loss to evaporation.

Fly consumption was documented by capturing digital images (JPEG format, 4000X3000 pixel resolution) of the setup at one-hour intervals for six hours. We estimated the amount of liquid consumed from each pipet over time after subtraction of liquid lost from the corresponding evaporation control pipet over the equivalent amount of time. Estimates of liquid volume were taken using the ruler tool in Image J version 1.47v (NIH- http://imagej.nih.gov/ij/). We measured the distances (in pixels) from the bottom capillary tip to the 5 µl reference line, and from the bottom capillary tip to the meniscus of the dyed liquid in the capillary, in each photograph. We converted the liquid length measurement to volume relative to the length measurement for the 5 *u*l reference line.

### Statistics

Analyses were conducted with SPSS software v. 20 (IBM corporation 2011).We analyzed fly longevity data using survival analysis with right-hand censoring of subjects that lived to the end of the study or were lost for reasons other than death. We tested for differences in suvival distributions [Pr(flies alive) versus insect age] using the log-rank (Mantel-Cox) test to make all pairwise comparisons among treatments within each experiment.

We tested differences among treatments in the percent of living flies that succeeded in the climbing assay on day seven using Fisher's exact test (two-tailed).

We used ANOVA to test for treatment and sex effects on consumption rates in the CAFE experiments. We calculated the total volume of fluid consumed by the flies in each capillary tube after subtracting the estimated amount lost to evaporation, then divided by the number of flies in each CAFE test chamber. We used the mean volume per fly as the response variable, and calculated this value for each hour of the trial; we treated mean consumption for each hour as a data point in the analyses.

## Results and Discussion

### Comparisons of effects of non-nutritive sweeteners

**Figure 1:**
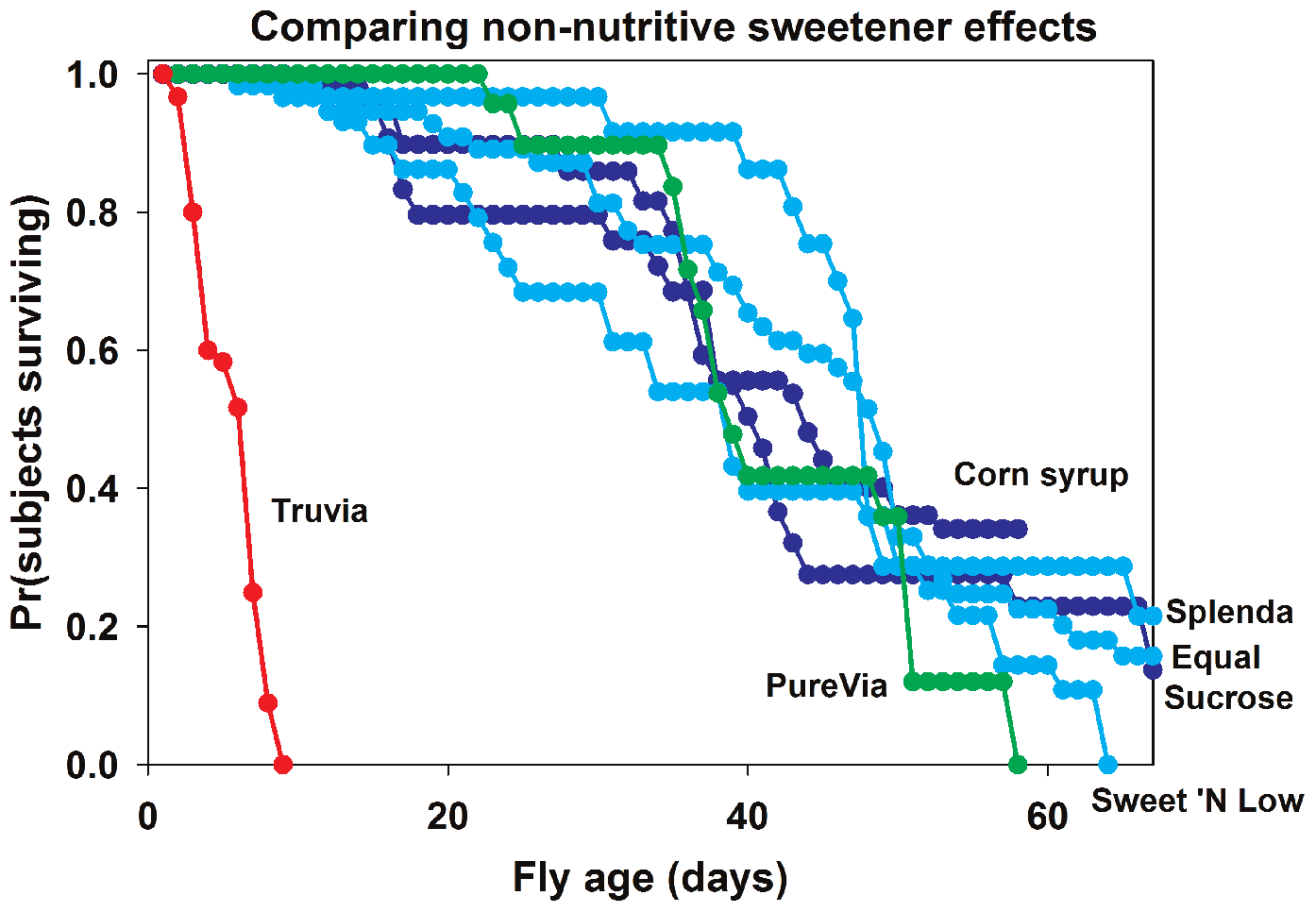
*Drosophila melanogaster* raised on food containing Truvia show decreased longevity. Truvia is red, Purevia is green, control nutritive sugars are dark blue, and other non-nutritive sugars are light blue. Graph shows percentage of living adult flies raised on food containing different nutritive sugars and non-nutritive sweeteners over time. Note significant decrease in longevity of adult flies raised on food containing Truvia compared to other food.

We initially compared the effects of adding five different non-nutritive sugar substitutes (Truvia, Equal, Splenda, Sweet'N Low, and PureVia; see Supplemental Figure S1 for the active non-caloric sweeteners and chemical structures in each sugar substitute) to standard lab culturing *Drosophila* food [6]. Adult flies raised on food containing Truvia showed a significant reduction in longevity (Figure 1, red line) compared to adult flies raised on control nutritive sweeteners (Figure 1, dark blue lines, both *X* ^2^>76.0; both *p<0.001*), Purevia (Figure 1, green line, *X* ^2^ = 76.3, *p<0.001*), and compared to other non-nutritive sweeteners (Figure 1, light blue lines, all *X* ^2^>73.0, all *p<0.001*). No other treatments differed significantly (all *X* ^2^<3.4, all p>0.06) except Splenda vs. Sweet ‘N Low (*X* ^2^ = 6.1, p = 0.01). While the mean longevity for flies raised on control and experimental foods without Truvia was between 38.6±3.2SE and 50.6±2.9SE days, the mean longevity of flies raised on food containing Truvia was 5.8±0.3SE days.

### Effects on motor coordination

**Figure 2:**
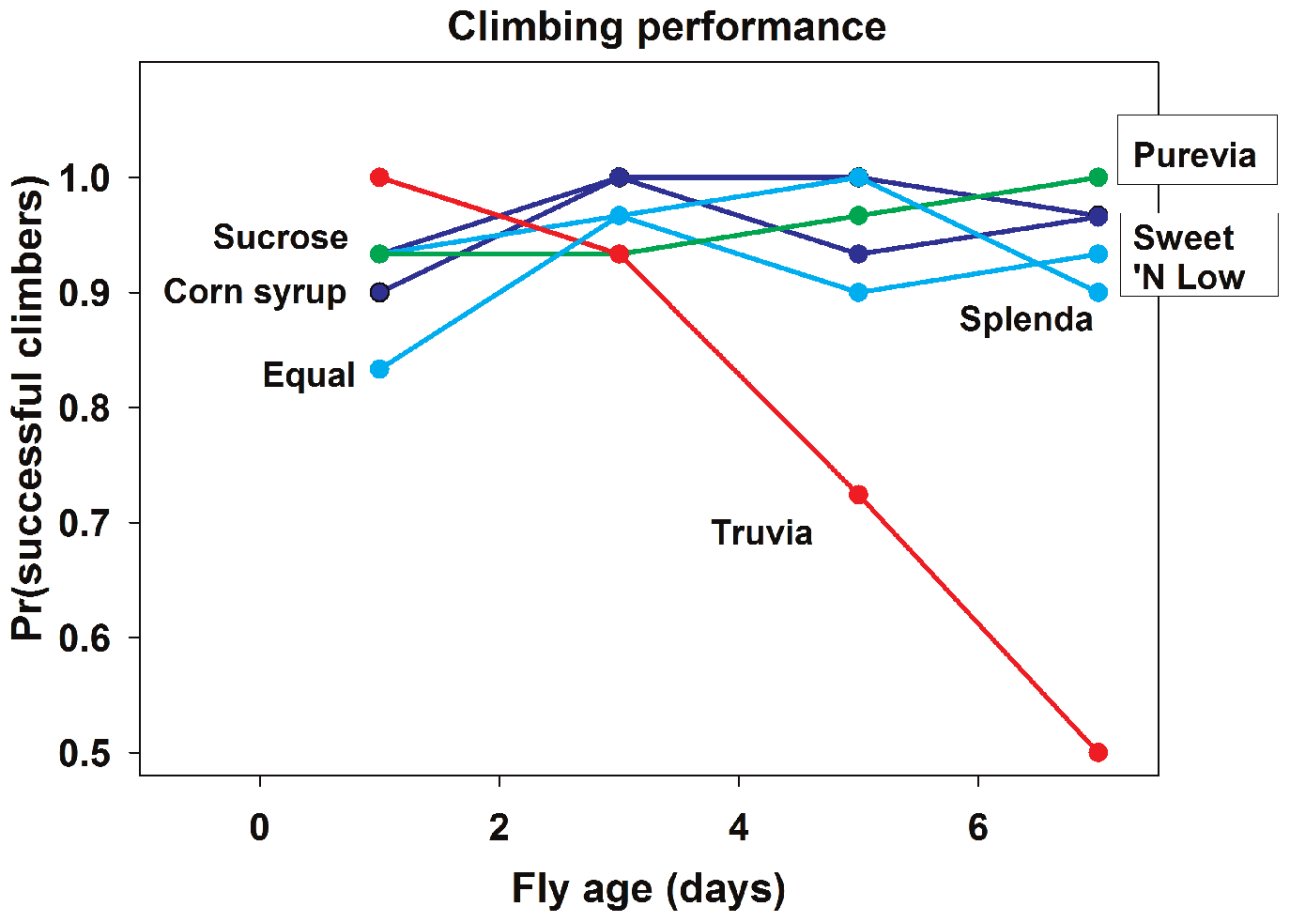
*Drosophila melanogaster* raised on food containing Truvia show decreased motor behavior. Truvia is red, Purevia is green, control nutritive sugars are dark blue, and other non-nutritive sugars are light blue. Graph shows climbing ability of adult flies raised on food containing different nutritive sugars and non-nutritive sweeteners over time. Note the significant decrease in climbing behavior of adult flies raised on food containing Truvia compared to other food.

We noted that adult flies raised on food containing Truvia displayed aberrant motor control prior to death. We therefore assayed motor reflex behavior through climbing assays. Flies raised on food containing Truvia showed a significantly decreased ability to climb by day 7 (Figure 2, red line) compared to flies raised on control nutritive foods (Figure 2, dark blue lines, Fisher's exact test, both *p = 0.0006*), Purevia (Figure 2, green line, *p<0.0001*), and compared to other non-nutritive sweeteners (Figure 2, light blue lines, all *p<0.007*). No other treatments differed from each other (all *p>0.24*). Taken together with our longevity studies, these data suggested some component of the non-nutritive sweetener Truvia was toxic to adult *Drosophila melanogster*, affecting both motor function and longevity of this insect.

### Tests of erythritol as the toxic agent

Our initial analysis of sweeteners included two sweeteners that contained extracts from the stevia plant, Truvia and Purevia (Figure S1). While adult flies raised on food containing Truvia showed a significant decrease in longevity compared to controls, this was not the case for flies raised on Purevia (both X^2^<1.1, both *p>0.30*, Figure 1). These data suggest stevia plant extract was not the toxic element in these sweeteners. Purevia contains dextrose as a bulk component, while Truvia contains erythritol as a bulk component. Erythritol is a four-carbon polyol that is marketed as a non-nutritive sweetener [10] (Figure S1). To determine if erythritol was the toxic component of Truvia, we repeated our longevity studies on food containing equal weight/volume (0.0952 g/ml) of nutritive sugar control sucrose, and non-nutritive sweeteners Truvia, Purevia, and erythritol. We assured the flies were successfully eating the foods containing these sweeteners through dye labelling the food with a non-absorbed blue dye [8] (blue food), and visual confirmation of blue food present in fly abdomens and proboscises daily (Figure S2). Most subject flies in all treatments had visibly blue abdomens throughout the study starting on day 1. The average percentage of blue abdomens throughout the study were 97.46% for Truvia, 98.46% for Purevia, 98.73% for erythritol, and 95.63% for sucrose. Importantly, some subjects were occasionally observed with no visible blue food in their guts followed by surveys when all flies contained blue food, suggesting guts were voided then refilled following ongoing consumption. These data confirm all treatment foods (including Truvia and erythritol treatments) were consumed by adult flies, and suggest mortality was not due to food avoidance and starvation.

**Figure 3:**
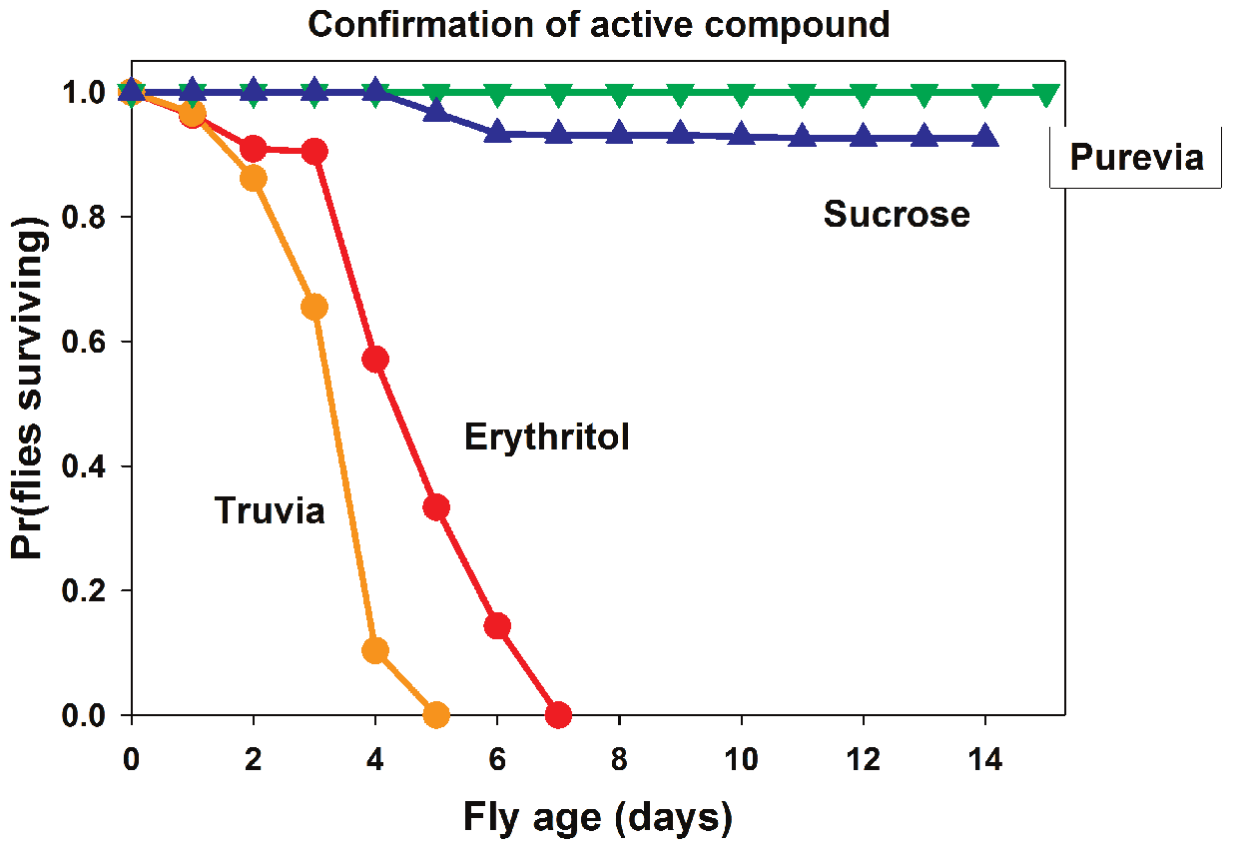
*Drosophila melanogaster* raised on food containing erythritol show decreased longevity. Truvia is orange, erythritol is red, Purevia is green, and the control nutritive sugar sucrose is dark blue. Graph shows percentage of living adult flies raised on food containing sucrose and non-nutritive sweeteners over time. Note significant decrease in longevity of adult flies raised on food containing either Truvia or erythritol compared to either Purevia or sucrose.

If Stevia plant extracts were the mortality causing agent(s), we expected both Truvia and Purevia to reduce fly longevity relative to the sucrose control. If erythritol was the mortality causing compound, we expected flies raised on Truvia and erythritol to have reduced longevity relative to Purevia and sucrose, which should not differ. Our data strongly implicated erythritol as the mortality causing agent within Truvia (Figure 3). Truvia-fed flies had shorter lifespans (mean+/−SE 3.60+/−0.17 days, Figure 3 orange line) than both Purevia-fed flies (*X* ^2^ = 61.46, p<0.001, Figure 3 green line) and sucrose-fed flies (*X* ^2^ = 60.05, p<0.001, Figure 3 blue line). For erythritol-fed flies, longevity (mean+/−SE 4.9+/−0.30 days, Figure 3 red line) was significantly shorter than both Purevia-fed flies (*X* ^2^ = 60.22, p<0.001) and sucrose-fed flies (*X* ^2^ = 51.88, p<0.001). Purevia and sucrose longevities did not differ (*X* ^2^ = 2.03, p = 0.15). Most flies in the Purevia (100% survival) and sucrose (93.3% survival) treatments were alive when observations were terminated at 15 days, so mean fly longevities could not be calculated for these treatments.

### Tests for sex differences in longevity when consuming erythritol

In order to determine if there were any sex related differences in longevity in response to the difference treatments, we compared the longevity of male and female flies. We found no consistent patterns of sex differences in longevity for flies consuming Truvia or erythritol (Table 1). Importantly, males and females never differed significantly in longevity for any treatment. In two experiments, the mixed sex vial differed from males only or from both pure-sex vials. In one case the mixed vial flies had shorter lifespans, in the other case the mixed flies lived longer. We conclude sexes did not differ in longevity responses to erythritol consumption.

**Table 1:** Tests for sex differences in survival of flies consuming erythritol.

Experiment/treatment	Mean longevity (days); Sexes being compared	Mantel-Cox test *X* ^2^ value	p-value
Sweetener comparison/0.24 g per ml Truvia	Male (5.75), Female (5.55), Mixed (4.90)		
	Male-Female	0.00	0.98
	Male-Mixed	**4.31**	0.04*
	Female-Mixed	2.94	0.087
Verifying active component/0.24 g per ml erythritol	Male (4.34), Female (5.33), Mixed (5.15)		
	Male-Female	2.27	0.13
	Male-Mixed	1.76	0.18
	Female-Mixed	0.24	0.62
Verifying active component/0.24 g per ml Truvia	Male (3.60), Female (3.16), Mixed (4.00)		
	Male-Female	1.15	0.28
	Male-Mixed	2.03	0.15
	Female-Mixed	3.82	0.051
Dose-response assay/0.1 M erythritol	Male (28.8), Female (29.0), Mixed (28.0)		
	Male-Female	0.013	0.91
	Male-Mixed	0.001	0.98
	Female-Mixed	0.007	0.934
Dose-response assay/0.5 M erythritol	Male (23.2), Female (21.8), Mixed (23.4)		
	Male-Female	1.86	0.17
	Male-Mixed	2.15	0.14
	Female-Mixed	0.18	0.89
Dose-response assay/1 M erythritol	Male (10.4), Female (9.7), Mixed (9.4)		
	Male-Female	0.16	0.69
	Male-Mixed	2.05	0.15
	Female-Mixed	1.71	0.19
Dose-response assay/2 M erythritol	Male (1.9), Female (2.0), Mixed (1.9)		
	Male-Female	1.00	0.32
	Male-Mixed	0.00	1.0
	Female-Mixed	1.00	0.32
Choice test/1 M sucrose vs. 1 M erythritol	Male (13.9), Female (12.4), Mixed (13.4)		
	Male-Female	3.07	0.08
	Male-Mixed	2.36	0.13
	Female-Mixed	0.22	0.64
Choice test/1 M erythritol vs. 1 M erythritol	Male (2.2), Female (2.3), Mixed (2.2)		
	Male-Female	0.25	0.62
	Male-Mixed	0.00	1.0
	Female-Mixed	0.25	0.62
Choice test/1 M sucrose vs. 2 M erythritol	Male (3.0), Female (2.9), Mixed (6.3)		
	Male-Female	0.16	0.69
	**Male-Mixed**	**9.64**	0.002*
	**Female-Mixed**	**12.52**	<0.001*

All experiments consisted of three vials of ten flies each: all males, all females, and mixed sex (5 males and 5 females). Under each experiment and treatment, the mean longevity in days for each vial is listed; mean longevities were calculated using Kaplan-Meir survival analysis with right censoring. Statistics for all pairwise sex comparisons are then listed (log-rank Matel-Cox test of survival distribution equality). Significant sex differences are listed in bold text and indicated by an asterisk.

### Dose-response analysis of erythritol effects on fly longevity

**Figure 4:**
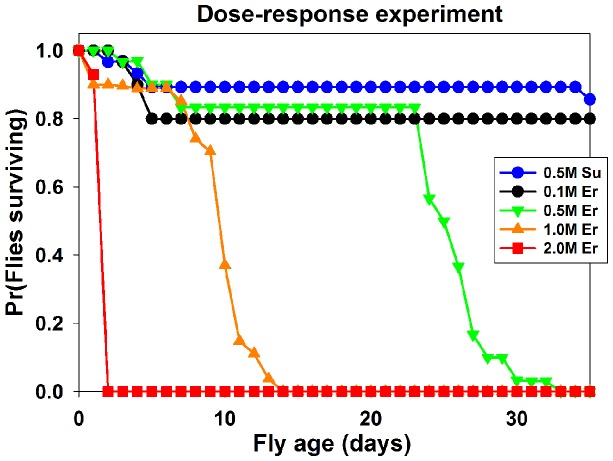
Increasing concentrations of erythritol show decreased longevity in *Drosophila melanogaster*. Graph shows percentage of living adult flies raised on food containing different concentrations of erythritol. Control food is 0.5(blue line). 2 M erythritol (red line), 1 M erythritol (orange line), 0.5 M erythritol (green line), and 0.1 M erythritol (black line) were used. Note significant decrease in longevity of adult flies as concentration of erythritol is increased.

Previous analyses were performed using equal weight/volume concentrations (0.0952 g/ml; approximately 0.78 M in the case of erythritol) of nutritive and non-nutritive sweeteners. To assess the utility of erythritol as an insecticide we repeated the longevity studies using erythritol at varying concentrations to determine erythritol's dose response. We assessed the effect of 0.1 M, 0.5 M, 1.0 M and 2.0 M erythritol-containing food on fly longevity. Adult flies showed a dose-dependent reduction in longevity when raised on food containing increasing concentrations of erythritol (Figure 4). Food containing 2 M concentrations of erythritol showed a significant and severe effect on longevity compared to all other treatments (all *X* ^2^>37.6, all p<0.001), although 1 M and 0.5 M also showed significant reductions in longeviety compared to flies raised on control food contatining 0.5 M sucrose (both *X* ^2^>42.1, both p<0.001). Flies fed 0.5 M erythritol lived longer than flies in the 1 M erythritol treatment (*X* ^2^ = 34.8, p<0.001). Flies raised on 0.1 M erythritol showed no significant difference in longevity compared to flies raised on control food when observations were terminated at 35 days subject fly age. Taken together, these data suggest increasing dosage of erythritol reduced fly longevity according to concentration.

### Palatability of food containing erythritol

Finally, to determine if erythritol-containing food was in some way repulsive to flies, we performed two different experiments: 1) two-way choice experiments, and 2) a CAFE experiment to directly test consumption.

**Figure 5:**
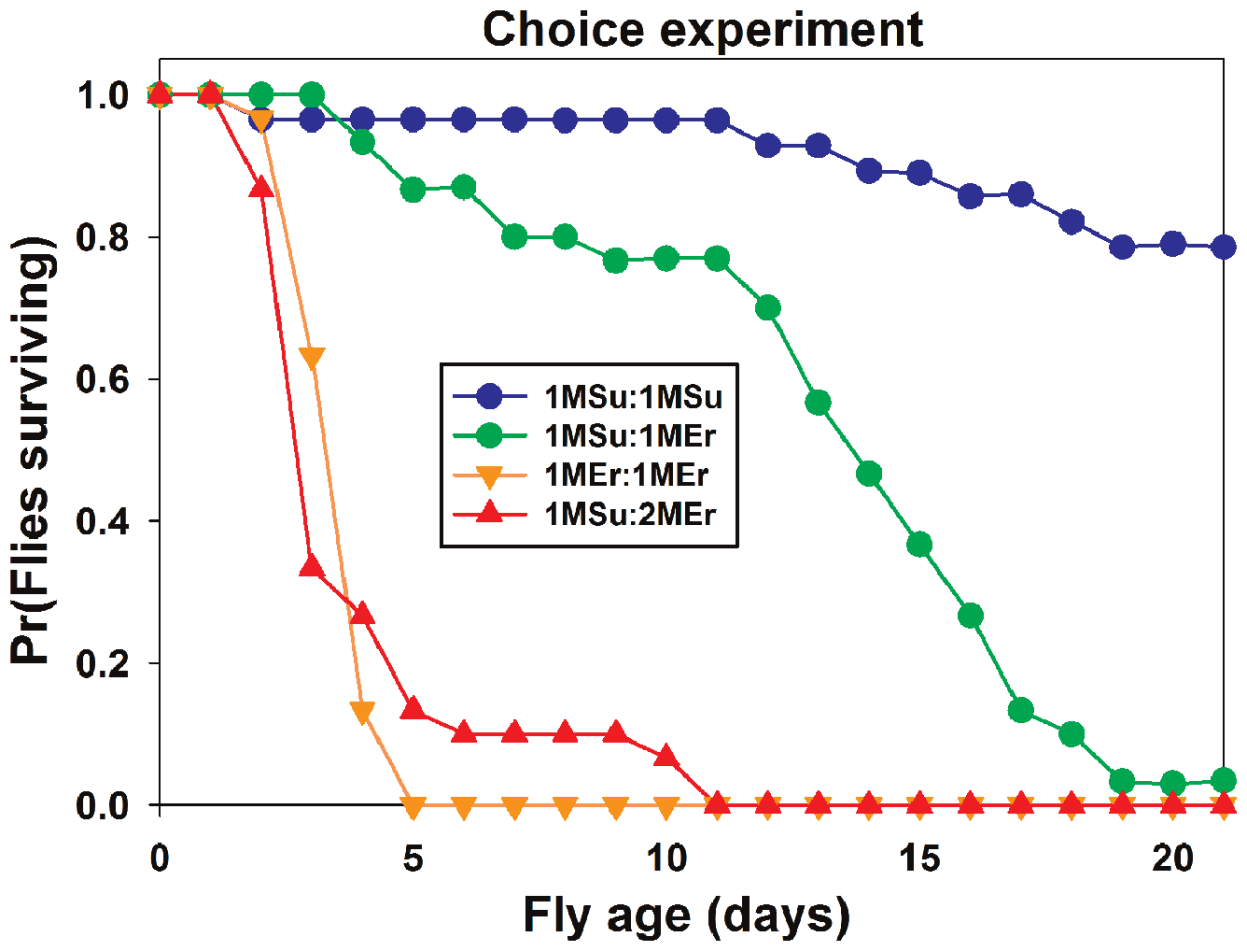
*Drosophila melanogaster* consume erythritol when given access to sucrose in a two-way choice experiment. Graph shows percentage of living adult flies when given a choice between two different food sources throughout their lifespan. Negative control choice experiments provide 1(blue line). Positive control choice experiments provide 1 M erythritol on both sides of the choice chamber (orange line). Experimental groups provide 1 M erythritol on one side of the choice chamber and 1 M sucrose on the opposite side of the chamber (green line); and 2 M erythritol on one side of the choice chamber and 1 M sucrose on the opposite side of the chamber (red line). Note significant decrease in longevity in both experiments where erythritol is provided as as a choice with sucrose.

First, we provided flies with free access to two food sources: 1 M sucrose control food, 1 M erythritol, and 2 M erythritol, and monitored their longevity over time. We used blue dye in one food per choice trial (Figure S3) to ensure that food was being taken up by the flies (see Materials and Methods). Flies with a choice between 1 M sucrose and 1 M erythritol had significantly decreased longevity realtive to sucrose:sucrose choice (*X* ^2^ = 37.5, p<0.001; Figure 5, green line). Longevity was also significantly reduced when we provided the flies with a choice between 1 M sucrose and 2 M erythritol (*X* ^2^ = 60.5, p<0.001; Figure 5, red line).

**Figure 6:**
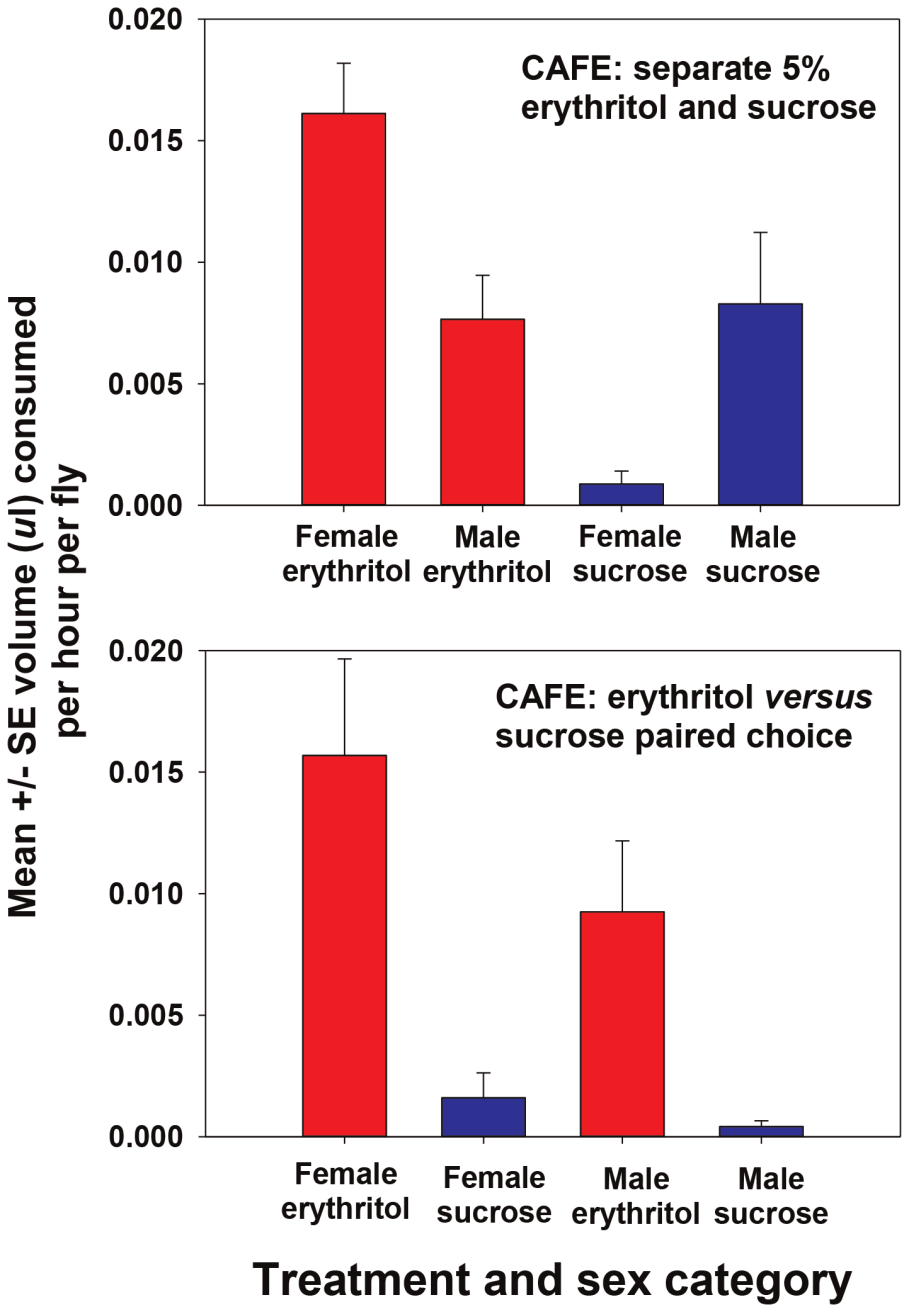
CAFE experiments show *Drosophila melanogaster* actively consume erythritol over time. Upper graph shows prandial behavior of 10 individually housed flies fed 5% erythritol (red colums) and 10 individually housed flies fed 5% sucrose (blue columns) over a 6 hour period. Average intake per fly per hour is graphed for each treatment and is separated by sex. Lower graph shows prandial behavior of 10 indivually housed flies when presented with a choice between 5% erythritol (red colums) and 5% sucrose (blue columns). Average intake per fly per hour is graphed for each treatment and is separated by sex. Note the significant increase in erythritol intake compared to sucrose intake for both sexes.

Second, to directly measure the amount of sugar consumed by flies over time, we performed CAFE experiments. In separate but simultaneous presentations of 5% w/v sucrose and erythritol we observed that female flies consumed erythritol at higher rates than sucrose, and male flies consumed erythritol at similar rates compared to sucrose (Figure 6; F_1,21_ = 12.90, p = 0.002). Sexes did not differ significantly in overall consumption rates (F_1,21_ = 0.07, p = 0.80), but males and females differed in their relative rates of consuming the two treatments (sex X treatment interaction term, F_1,21_ = 15.23, p = 0.001).

When given a choice between 5% w/v sucrose and erythritol in each chamber, flies again consumed erythritol at higher rates (Figure 6; F_1,21_ = 20.51, p<0.001). Sexes did not differ significantly in overall consumption rates (F_1,21_ = 2.30, p = 0.15) and males and females showed similar patterns of consumption across treatments (sex X treatment interaction term, F_1,21_ = 1.08, p = 0.31).

Taken together, the data from these two experiments show that flies consumed foods containing erythritol when given access to sucrose-containing (control) food. Further, our CAFE data suggest that flies actively choose erythritol over sucrose when presented with both. We conclude erythritol baits could function as an effective insecticide delivery mechanism when presented in naturalistic situations where insects have access to other foods.

## Conclusions

Our findings demonstrate, for the first time, that erythritol, and the erythritol containing sweetener Truvia, are toxic to *Drosophila melanogaster*. Our studies did not address the physiological or molecular mechanisms of erythritol toxicity. In some insects, ingested erthritol can inhibit uptake of nutritive sugars through the gut wall [11]. Ingestion of erythritol may alter nutrient and/or water absorption and/or efflux.

However, erythritol in tissues is not always toxic to arthropods. For example, some insect species that are seasonally exposed to freezing conditions produce erythritol and other polyhydric alcohols as tissue cryoprotectants [12], [13]. Larvae of one antarctic midge can safely ingest erythritol from food plants and sequester it for adult cryoprotection [14]. Toxic effects of ingested erythritol may be dose-dependent, as our data suggest.

Of note, erythritol is not the only sweetener known to be toxic to insects. For example, mannose has been shown to be toxic in honey bees [15], [16]. However, mannose was not toxic to *Drosophila melanogaster* or to *Ceratitis capitata* [15]. Further study will be required to determine if erythritol is toxic to other insect species.

A large body of literature has shown that erythritol consumption by humans is very well tolerated [5], [17]–[19], and indeed, large amounts of both erythritol and Truvia are being consumed by humans every day throughout the world. Taken together, our data set the stage for investigating this compound as a novel, effective, and human safe approach for insect pest control. We suggest targeted bait presentations to fruit crop and urban insect pests are particularly promising.

## Supporting Information

Figure S1 **List of artificial sweeteners, active compounds, and structures of active compounds for each sweetener used in this study.** Panels show list and structures of each sweetener used in this study.
(TIF)

Figure S2 **Blue food labeling show** ***Drosophila melanogaster*** **eat food containing Truvia and other non-nutritive sweeteners.** Panels show representative female (A) and male (B) flies with blue abdomens and proboscises (arrows in panels A and B).
(TIF)

Figure S3 **Schematic representation of food choice trials performed.** Panels show schematic of the presentation of food choice trials between erythritol and sucrose.
(TIF)
