# Supplementary figures and images for: Erythritol, a Non-Nutritive Sugar Alcohol Sweetener and the Main Component of Truvia®, Is a Palatable Ingested Insecticide
Source: PLoS One. 2014 Jun 4;9(6):e98949. doi: 10.1371/journal.pone.0098949 (PMC4045977; doi:10.1371/journal.pone.0098949)

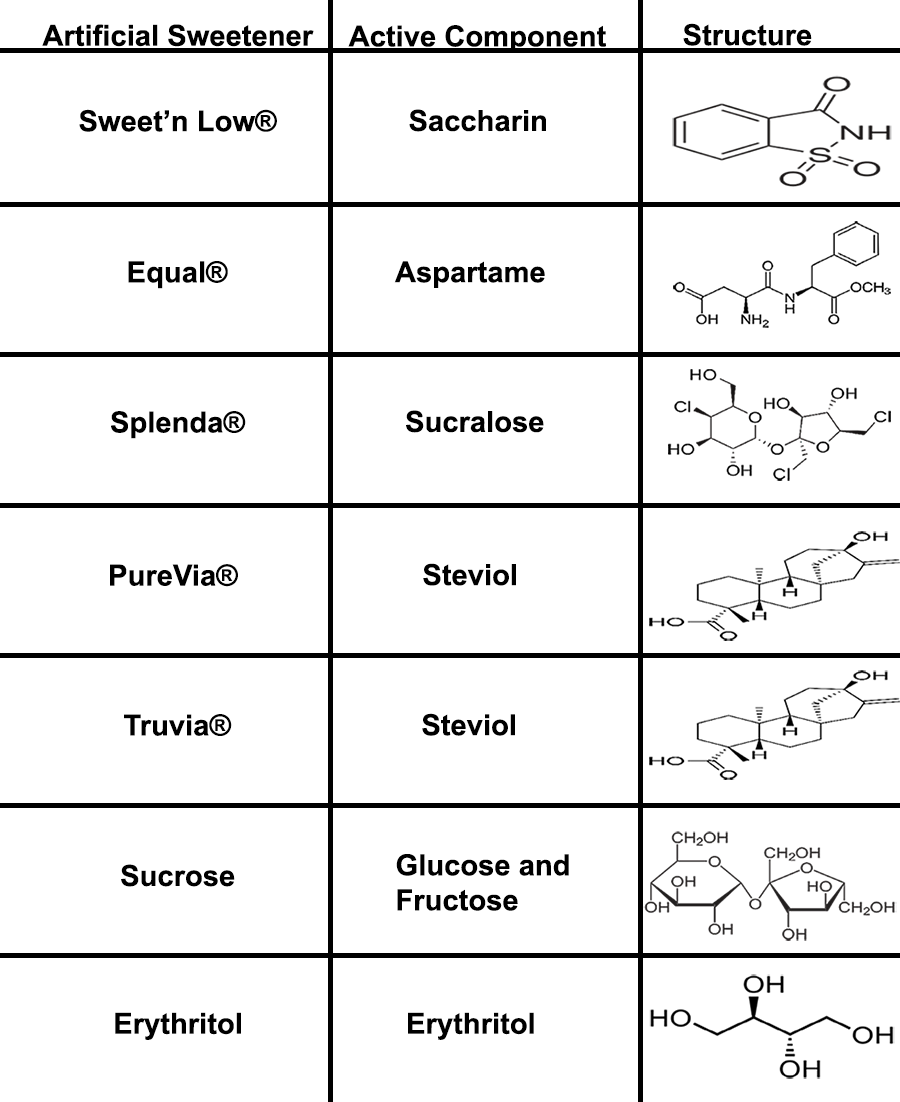

Supplement: Figure S1 — List of artificial sweeteners, active compounds, and structures of active compounds for each sweetener used in this study. Panels show list and structures of each sweetener used in this study. (TIF) [file pone.0098949.s001.tif]

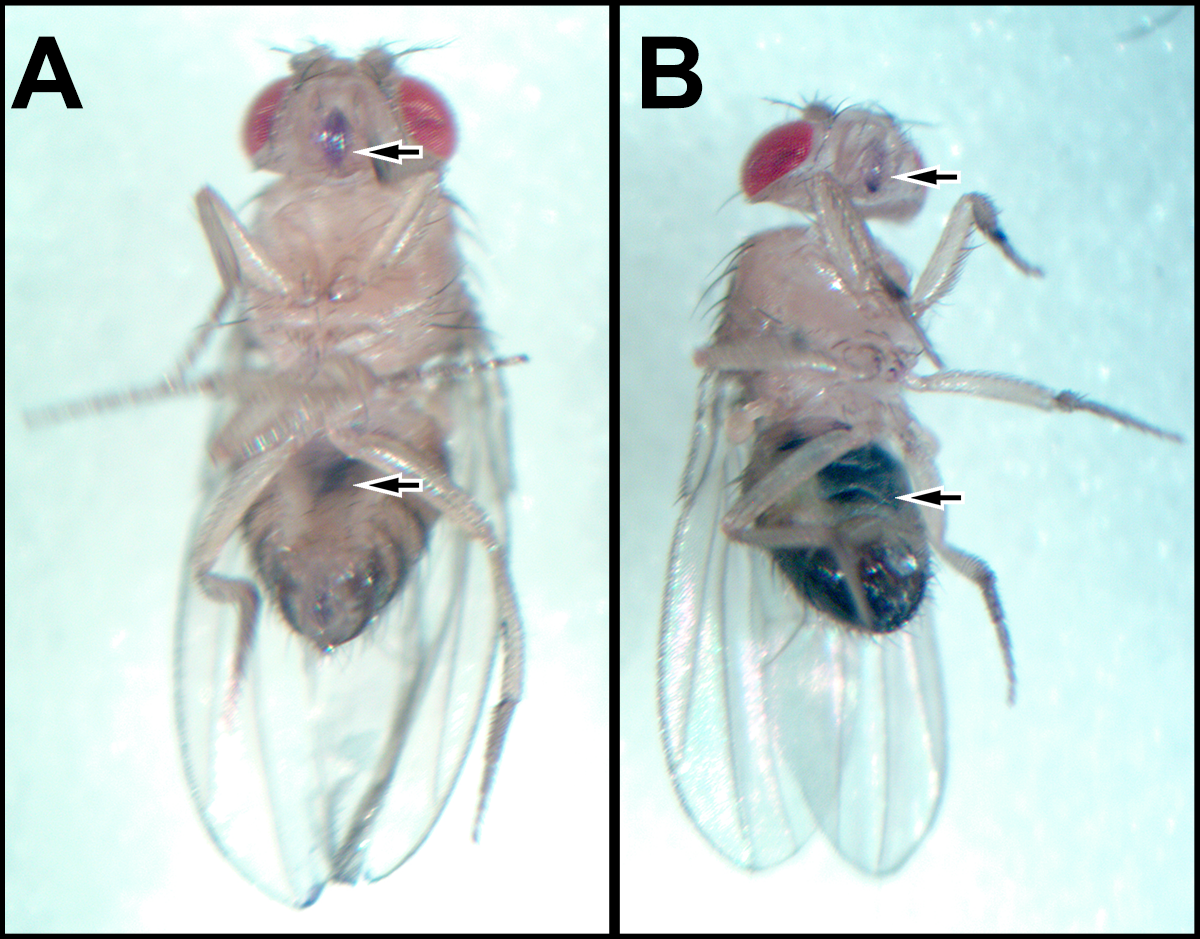

Supplement: Figure S2 — Blue food labeling show Drosophila melanogaster eat food containing Truvia and other non-nutritive sweeteners. Panels show representative female (A) and male (B) flies with blue abdomens and proboscises (arrows in panels A and B). (TIF) [file pone.0098949.s002.tif]

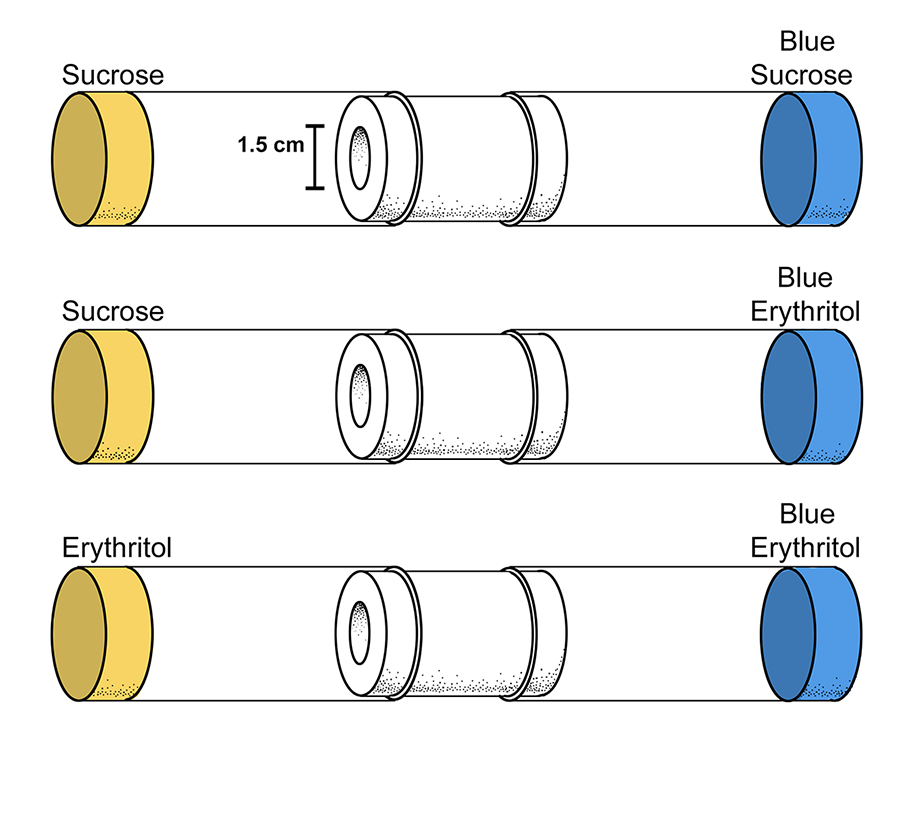

Supplement: Figure S3 — Schematic representation of food choice trials performed. Panels show schematic of the presentation of food choice trials between erythritol and sucrose. (TIF) [file pone.0098949.s003.tif]
